# The Art of Medical Diagnosis

**Published:** 1845-10

**Authors:** 


### ART. II.

*Technik der Medicinischen Diagnostik.* Von Dr. A. SIEBERT, &c. Erster Band.—*Erlangen*, 1844.

*The Art of Medical Diagnosis.* By Dr. A. SIEBERT, &c. Vol. I.—*Erlangen*, 1844. 8vo, pp. 408.

MEDICAL diagnosis is the art of discovering the nature of diseases and of distinguishing them from each other. The right practice of it requires, firstly, a knowledge of the human body in a healthy state, the position, size, and structure of its various organs, their functions, and their anatomical relations. The practitioner must also be acquainted with all known morbid conditions, and be capable of deducing from the data of pathological anatomy and physiology, all possible departures of the human organism or of its organs from the normal condition. These are Dr. A. Siebert's views.

As general principles, we readily admit their correctness. But there is another kind of diagnosis of humbler pretensions, but perhaps not less useful than the scientific and recondite art discussed by the author of the volume before us, and we are quite sure, extensively practised. This is the method of empirical diagnosis. We term it empirical, because it is conversant mainly with experience. The science of medicine being confessedly imperfect, so also must be scientific diagnosis. We do not know the nature of many or most diseases; but we have learnt the lessons of experience, and therefore in practice it is, that our diagnosis is empirical. The practitioner in consultations quotes a "case" to his medical brother; or in daily practice quotes it to himself. The nature of the case he perhaps only guesses at; the remedies which he administered successfully he knows well. In consulting the written experience of his predecessors on any particular case, the treatment of which is rebellious, the diagnosis of the practitioner is also empirical. He is more anxious to find out an instance like his own, so that he may treat it empirically, than to seek for general principles or make logical deductions. This, by way of introduction, and as a hint to future writers on diagnosis.

Scientific diagnosis is made up of several subdivisions. First, is *anamnesis;* this comprises (in one short sentence) the medical biography of the patient. It ascertains his physiological relations, the external and internal causes of disease, their origin and mode of action, and the order of development of the morbid phenomena. Without this anamnestic inquiry, the true condition of the patient at the time of inquiry (the *status præsens*) can only be imperfectly ascertained. *Symptomatology* is another division of diagnostics. It considers all the changes in the structure and functions of organs from the healthy condition, and their relation to the disease; and it includes the art and mode of ascertaining these deviations, namely, the methods of exploration. *Semeiology* interprets and estimates the symptoms, endeavours to apply special nosology and pathological anatomy to their elucidation, and harmonizes them with the facts of general anatomy and physiology. Compounding or composite diagnosis arranges the facts thus worked out—the *signs* of diseases—into groups, according to physiological and pathological principles, and brings these groups into general relation, so as to develop a special form of disease, which it is the duty of comparative diagnosis to compare with the ideal representative of special nosology deduced from observation and experience. *Prognosis,* the last division mentioned by Dr. Siebert, pronounces as to the further course and termination of the series of morbid phenomena. The following extract will illustrate our previous statements:

"These divisions into which diagnosis resolves itself correspond to the following questions, the answers to which it is the physician's business to ascertain.

"1. What led to the disease? why, through what agencies, and in what manner did the sickness happen?

"2. What aberrations from the normal condition of the organs are to be observed?

"3. How are these aberrations to be explained, and what they themselves signify?

"4. How are they related to each other? what is their relative value, and how are they to be made a whole so as to constitute a specific disease?

"5. What are the grounds for deciding that it is *this* disease and no other?

"6. What will be its course and termination?" (p. 56.)

Dr. Siebert takes up the different divisions of his subject in succession, under the head of *general diagnosis* and *clinical propædeutics.* About two hundred pages are, however, devoted to the means and method by which the patient should be examined.

It is manifest, that skill in diagnosis is very much dependent upon the personal qualifications of the physician. Can he be a good auscultator without, what the musicians term, an ear? or can he have delicacy of touch without the power to perceive minute shades of difference in the form and resistance of structures? or can he perceive instructive analogies quickly if he have not a good memory, and that invaluable mental power by which observations are tabulated in the mind as quickly as they are perceived? moral qualifications are also necessary. The physician who would be a good diagnosist must not be indolent or careless; must avoid the extremes of too great kindness or severity; must be neither too grave nor too gay. Tact should also be observed in examining a patient. The physician should take care to have him in a good light. His temper, habits of life, manner of speaking, the state of the room in which he reposes, in fact, everything should be scrutinized in a quiet, placid manner, removed alike from taciturn severity, and garrulous abruptness. Twenty-five rules of conduct are laid down by Dr. Siebert for the practitioner, the propriety of which cannot be questioned. It is probable, however, that one good example would avail more in teaching than a folio volume of rules. It is marvellous how little the *lex scripta* of medicine is heeded.

*The methodical examination of a patient.* Three methods are discussed: firstly, the synthetical, genetical or historical; secondly, the analytical; and thirdly, a method composed of the two. The first begins the inquiry with the birth of the patient; makes reference to the health of his ancestors or relatives, and traces hereditary diseases; passes in review the diseases of infancy; inquires into his rank and occupation, his bodily constitution, usual morbid states, mode of living, habits, inclinations, and idiosyncrasies. This step being taken, the physician next ascertains the causes of the existing affection, the mode of its commencement, its course, and the operation of the remedies already used. The *anamnesis* being completed, the examination of the *status præsens* is proceeded with. In this, the inquiry as to the state of the organs and of their functions, is carried on in a systematic and regular manner.

The synthetical method is directly opposed to the analytical. It commences with the status præsens of the patient, and endeavours to ascertain the principal derangement of structure and function, and its relations. From hence it conducts the anamnesis by retrospective process.

The third method, compounded of the preceding, is the most useful and most generally applicable, for while it secures the advantages, it avoids the disadvantages of the others. It collects the retrospective facts only which bear on the case, and affords the patient an opportunity of making his statements in a historical sequence. The synthetical method is the most scientific and thorough. Dr. Siebert transcribes the questions and answers in a case of "enterohelcosis" (ulceration of the intestines) with perforation, examined by him after this method; it is an interesting example of clinical research, extremely instructive, and creditable, we think, to its author.

*On the discovery of the objective phenomena of disease, and on the*

*methods of exploration in general.* Those phenomena are objective which the physician observes himself; those subjective which he can learn from the patient only. In ascertaining the former, several points are worthy of notice. First, the regions of the body require to be defined, and the relative position of the corresponding organs. Dr. Siebert discusses the regions defined by Raciborski, Velpeau, Borck, and Williams, and gives a lithographic sketch of those which he himself adopts, naming them after Raciborski's method. These are illustrated by two plates; but as the slight alterations introduced by the author add little or nothing to our previous knowledge of the subject, we avoid entering into details.

*The art of measuring.* Under this head, Dr. Siebert gives instructions for the admeasurement of the skull in its various diameters, of the thorax in every way, and of the abdomen. Dr. Siebert objects to the plan of Montault which unites the measuring tape with the stethoscope, as impairing the utility of the latter. Among the instruments referred to, or described, is Canstatt's pnoiometer. This consists in a slip of parchment, an inch in breadth and 110 to 120 centimetres (36 to 40 inches) in length, furnished at one end with a fastner like that on pocket-books. To measure the dilating force of the thorax, the measure is put round the chest, the end slipped through the slider or bar, and then it is drawn as tight as possible. The patient now breathes deeply a few times, and the difference before and after inspiration expresses in centimeters the degree of dilatation of the thorax during inspiration.

*The art of percussion.* Dr. Siebert thinks the wooden or caoutchouc pleximeters have no superiority over the old ones made of ivory, or over the finger. Special directions are given as to the art of percussion so as to elicit correct sounds, an art by no means generally understood. Dr. Siebert knows physicians, who year after year, percuss with the wrist immoveable, with their nails, and fingers improperly applied, &c., and who rarely, if ever, elicit correct sounds. Eight fundamental sounds, each having three variations, are described by our author, and those of Piorry and Roda, Briançon, Williams, and Raciborski, are discussed.

*The art of auscultation.* Of this there are three kinds, the mediate, immediate or direct, and the *auscultatio ad distans.* The whole chapter forms a complete monograph on the structure and use of the stethoscope.

*The application of chemistry, and of the microscope, to diagnosis.* These two chapters are devoted to a description of the chemical and microscopical apparatus necessary to chemical research, and contain minute rules for their proper use. The late Dr. I. F. Simon contributes the materials of the chemical chapter.

*On the external exploration of the patient and the results.* The first point to be ascertained is the state of nutrition generally. Corpulent individuals, for example, are more subject to inflammatory affections, epidemical diseases, and sudden death, than those who are lean. Emaciation is more frequently a consequence of disease than obesity. The latter is, however, observed in polysarcia and chlorosis gigantea. Sudden emaciation is a *malum signum* in acute diseases, indicating a deep-seated lesion of important organs. When it accompanies diarrhœa from probable intestinal ulceration, or chronic vomiting, or the symptoms of acute hydrocephalus, the prognosis is gloomy. On the other hand, rapid wasting in typhus or scarlet fever, or in the "morbus maculosus," it is by no means a fatal sign. We have learnt by repeated experience that the patient may be so prostrated as to present an appearance not much unlike the facies hippocratica, and recover speedily. The emaciation of special parts of the body is observed in some diseases. In disease of the liver or spleen, the face and extremities first waste, in tubercular phthisis, the upper extremities and clavicular regions.

*The form and size of special organs* is another point to be ascertained from an external examination of the patient. Thus, the skull may become generally enlarged, or certain points developed, the outlines altered from the normal condition, &c. So also changes dependent upon constitutional predisposition or actual disease may be observed in both the hard and soft parts of the neck, thorax, abdomen, and limbs.

*The temperature* is another point to be carefully attended to; under this head Dr. Siebert gives a general outline, of the physiology and pathology of animal heat so far as they bear on diagnosis.

*The state of the cutaneous exhalation* presents numerous points of value in diagnosis. It is dependent on the condition of the cutaneous structures themselves, or of the state of the surrounding medium, on the activity of the internal secretions, on the rapidity of the circulation, and the condition of the blood. The average quantity of fluid transpiration is estimated at 29 ounces daily, containing from 7 to 8 scruples of solid matter. Children perspire more profusely than adults, men more than women. Men with fine hair and fair complexion readily perspire; and they have a predisposition to rheumatism and gout. Local sweats have considerable pathological importance. Perspiration with burning heat on the cheeks and nose is a sign of pharyngeal or gastric irritation; on the chest of structural change in the lungs. Local sweats are sometimes critical, as of the feet, when the excretion has an odious stench. We have seen a case of this kind, in which the sweat stood in drops on the feet, fresh drops springing up as fast as the feet were wiped. And it was curious that the surface affected occupied the posterior half only of each sole.

The connexion of the cutaneous exhalation with internal organs is important. The mutual dependence of the skin and kidneys is very obvious, not so also that between the lungs and the skin. Dr. Siebert observes that they suffer when the cutaneous transpiration is both diminished and increased. In the former case, the amount of pulmonary transpiration is morbidly increased; in the latter it is diminished, while the function of the lungs is exalted to meet the waste of oxygen through the skin. Profuse perspirations artificially excited, as in the "water cure" are extremely injurious to these organs. When there is a predisposition to pulmonary hemorrhage or tuberculous deposit, they are readily developed by this method of treatment as by every other cause of increased thoracic action. An extensive burn proves fatal in the same way. The functions of the skin being arrested, those of the lungs are morbidly exalted. In phthisis (Dr. Siebert argues) the colliquative sweats and diarrhœa are in like manner dependent upon the arrest of function in the lungs.

Dr. Siebert notices the various pathological phenomena implicating the cutaneous secretion at length. A whole section is devoted to the electric tension of the skin. The results of experiments on this point are however contradictory. Pfaff and Ahrens found in general that the skin was positively electrical in health; that men of a sanguineous, gave out more free electricity than those of a phlegmatic temperament; that there is more in the evening than in other hours of the day; that spirituous drinks increase the quantity; that women are more frequently negatively electric than men, and in the negative condition more particularly during pregnancy and menstruation; that a cold surface gives out no electricity; and lastly, that during the progress of rheumatic affections the amount falls to zero, from which it gradually rises as the disorder departs. Buzzorini found that cholera patients gave out electricity during the crisis of the disease. In scarlet fever and analogous affections, the gold leaves of the electrometer diverge much more actively than in health, and if lycopodium seeds be strewn on the skin, and then blown off they form into tolerably regular figures. Experimenters on pathological electricity are, however, far from being agreed, although the better authorities are in favour of the facts stated. The electric condition of a patient has an important relation to that of the earth and atmosphere. Dr. Siebert discusses the ætiological influence of exoteric electricity; a subject important in itself, but having no direct connexion with diagnosis.

*The colour of the skin* presents many pathological varieties of importance in diagnosis. Under this as well as the previous heads, no reference is made to the diagnostic value of symmetrical or asymmetrical phenomena. This is an omission Dr. Siebert will do well to fill up. Dr. Laycock, Dr. Budd, and Mr. Paget are the most recent writers on this subject.

The position and movements of the body and limbs, and the constitutional peculiarities and physiognomy of the patient are of importance in all their details to a correct diagnosis. It is from these indeed that the physician possessed of quick perception and tact derives his most valuable information. Dr. Siebert distinguishes six groups of physiognomonical rugæ. The *R. transversæ,* situate in the forehead, formed by the frontal muscle, express excessive pain arising externally. The *R. oculo-frontales,* extending from the forehead vertically to the root of the nose, express distress, anxiety, anguish, and severe internal pain. They also indicate in acute diseases an imperfect or false crisis, an impending efflorescence, and often a fatal termination. In severe headache, both the classes of rugæ just mentioned are observed. When the former join the latter abruptly in a disease, paralysis is impending or commencing. The *linea oculo-zygomatica* (of Jadelot) extending from the inner angle of the eye somewhat below the cheek-bone indicates in children a cerebral or nervous affection; in adults, disorder or abuse of the generative organs. The *linea nasalis* of Jadelot and De Salle (the Rhinal-linea orbicularis of K. H. Baumgärtner) begins at the upper border of the ala nasi, and extends more or less curved to the outer margin of the orbicularis oris. It is strongly marked in phthisis and atrophy. The inferior portion (*linea buccalis*) indicates gastric disease; the upper portion (the proper *linea nasalis,*) marks an affection of the upper part of the intestinal canal. Occurring conjointly with retraction of the cheek and with the *L. oculo-zygomatica,* the eyes being fixed and the complexion wan, it is a certain indication, according to Pieper, of worms. The *L. labialis* extends from the angle of the mouth, and is lost in the lower part of the face. In children it generally marks a thoracic affection, which renders the respiration laborious or painful. The *L. collateralis nasi* passes downwards in a semi-circular direction towards the chin, and externally to the *Linea nasalis, buccalis,* and *labialis.* It generally indicates chronic and obstinate disease of the thoracic or abdominal viscera. Dr. Siebert gives a similar account of the symptoms indicated by the mouth and eye. It is manifest that no verbal description can convey a true idea of their value. We have often wondered why in this age of pictorial literature no one has given a series of lithographic drawings, exhibiting the external and visible symptoms of disease.

Several morbid constitutions are considered by our author. The cerebral, apoplectic, medullary (spinal), plethoric, pulmonary, phthisical, abdominal, hepatic, splenic, arthritic, onanistic, and the tippling under the varieties of the wine, spirit, and beer tippling, are all noted in detail. Some of these are, we think, ill defined, others badly described. The arthritic constitution, the proper diagnosis of which is of very great practical importance, is dismissed in six or eight lines; without in fact indicating its true characteristics, which are those subsequently noted as proper to the arterial constitution. The hemorrhoidal habit, not generally recognized by British practitioners, and corresponding to Dr. Siebert's venous constitution, is characterized by the predominance of the organs in connexion with the portal system. The parts of the body below the diaphragm are sensibly more developed than in the normal condition. In the more exquisite forms, the pelvis and lower extremities are proportionately larger than the thorax and upper extremities. The skeleton of hemorrhoidal men approaches in form to that of the female. The veins are also more developed than the arteries. In the cheeks, nose, and the conjunctiva (the recognized varicose hemorrhoidal eyes) there are isolated and star-like groups of injected blood-vessels.

The physiognomy and constitution induced by specific forms of disease are next noticed. That significant of cardiac disease comes first: and Dr. Siebert makes the remark—very important if true—that softening of the heart is frequently the cause of mental derangement, which is mistaken for hypochondria.

The scrofulous constitution is considered under two forms, the irritable and the torpid. The first is seen in children with delicately formed extremities, delicate velvety skin, brown complexion, and dark hair. The eyes are dark and brilliant, the lashes long, the lineaments of the face finely drawn and expressive, the mind precocious. The torpid is connected with a fair complexion, thick and swollen nose, chin broad, the teeth irregular, late developed, and early becoming yellow and carious. Inflammation of the meibomian glands, scrofulous ophthalmia, intolerance of light, eruptions on the head, nose, lips, enlarged cervical glands, &c., accompany this well-known scrofulous diathesis. We subjoin Dr. Siebert's description of the scabious or itch-constitution, as one not generally recognized in England.

"The itch-constitution is frequently observed many years after the itch has been extirpated from the skin, without the itch-disease being cured; more seldom, however, when the itch passes by metastasis into some distinct form of internal disease than when breaking out from time to time on the skin, it returns again to the latent state, and exhibits its peculiar phenomena in an erratic manner. I have remarked the itch-constitution in many individuals, and known and treated it as such before its existence was shown by examination and inquiry, more particularly in journeymen mechanics infected in their travels, and with whom the itch had lingered for years. It was manifest by the puffed-up, swollen, plump face; the smutty eyes; the dull silly stare; the swollen nose and lips, the latter of a sallow hue; the puffy eyelids; and the general dull tint of the skin, when none of the usual complaints of vertigo, debility, indigestion, shortness of breath, lassitude, and sleepiness, had given indication of the disease. Many subsequently showed the truth of the early diagnosis by an itch-eruption more or less copious. Some became phthisical, others epileptic. In one case, the fits of vertigo increased in intensity, until the patient was insensible in them: these terminated by a discharge of a limpid fluid from the nostrils. In another case, death followed a remarkably acute attack of hydrothorax." (p. 198.)

We are not quite sure that British practitioners are right in slighting the doctrine generally received in Germany as to the constitutional origin of the itch. Cases, apparently of this kind, occasionally occur in families of obstinate cutaneous diseases highly contagious (scabies cachectica), characterized by the larger pustules and vesicles of scabies, on the hands and feet, and by minute pustules on the neck, shoulders, axillæ, and extremities, ending in leprous-like patches varying in size from a fourpenny-piece to half-a-crown. Sometimes patches of psoriasis are intermingled. The cure is difficult on account of the repeated relapses to which the individuals are subject. After the eruption has apparently disappeared, fresh pustules appear on the shoulders, scattered here and there, then extend to the axillæ, then over the deltoid muscle, &c., and terminate in the round leprous patches described.

Dr. Siebert refers to that rapidly fatal form of "gastrobrosis," the circumscribed perforation of the stomach, almost peculiar to young women, and describes its physiognomonical characters, but he gives us no means of anticipating its probable occurrence, nor does he even allude to the fact, well known in England, that it principally attacks the sex.

*Anamnesis.* Under this head Dr. Siebert considers the art of diagnosis as connected with the age, sex, race, and nation, family predisposition and hereditary peculiarities, education, domestic and civic life, trade and occupation, diet, dwelling, epidemic influence, temperaments, previous diseases, &c. We can only notice—

*Hereditary predisposition.* There is no subject connected with medicine which would more amply repay a scientific investigation than this. The facts are numerous, and can be arranged on an irrefragable general principle, namely, that every portion of the body of the offspring participates more or less in the physiological conditions of the parents. The first step in the inquiry would be, to ascertain the physiological laws of hereditary transmission. The true direction in which to seek this, is in embryology, and particularly in the development of tissues. Dr. Siebert remarks, to illustrate our views, that the gout of the parents may appear in the offspring as stone, or disease of the heart. This we know to be true, and the explanation, we conceive, is, that the serous layer of the embryo has received the hereditary predisposition of the parent. This predisposition is shown in a tendency to disease of the embryonic products of the serous layer, namely, the proper serous membranes and the bones and muscles with their appendages, namely, sero-synovial sacs, tendons, cartilages, &c. With this predisposition a morbid action in and a metamorphosis of the serous tissues is most frequent. Thus, in persons of this constitution, the muscular and tendinous fibres, and the cartilages, will change into bone—the serous membranes into cartilage—while the sub-serous membranes of the hollow muscles become the nidus of ossific deposits. Dr. Siebert remarks that gonorrheal fathers have scrofulous rachitic children. In this example, the mucous layer of the embryo and the mucous surfaces and tissues derived therefrom have the morbid predisposition of which the gonorrhea and the scrofula are special manifestations.

Special organs suffer also hereditarily, just as minute personal traits are transmitted to offspring. M. Boucher inquired into the fate of 58 individuals, the offspring of 14 epileptic females. Of these 32 died of convulsions in infancy, and of the 26 remaining, 7 had different nervous affections, 2 convulsions of an intermitting type, 2 were epileptics, and 1 hysterical; so that 14 only of 26 who grew up, remained free from disease of the nervous system. As we shall revert to this important question elsewhere, nothing more need here be said, except that Dr. Siebert's discussion of the subject is superficial and contains nothing novel.

We pass over the subjects already indicated without comment, to note Dr. Siebert's views of the epidemic constitution of the year; a subject truly important to be known in the diagnosis and treatment of disease. Dr. Siebert justly ridicules the partial views of those pathologists who see an inflammatory, a rheumatic, a catarrhal, gastric, nervous, &c. genius according to their preconceived notions; he is only surprised that they have not discovered a sanguineous, or osseous, or serous constitution. The fact most generally recognized, is that the predominant constitution or genius attracts all other diseases to itself, and impresses upon them its own type. But what is the cause of the predominant constitution? Dr. Siebert, in common with the majority of enlightened practitioners, looks for it in the meteorological changes proper to climates and seasons. Different climates have each their permanent "constitutions,"—so have the seasons. A cold, dry winter is as assuredly marked by inflammatory affections of the lungs, as by depression of the thermometer. Dr. Siebert enumerates several analogous instances of the seasonal recurrence of disease. The truth we think is this, that the meteorological changes determine a predominance or cessation of action in special organs, and it is these functional changes that determine the epidemic constitution just as they determine the individual constitution. Only in the latter case the functional activity or repose is permanent or alters only with age; in the former it alters with every great meteorological change.

*Composite and comparative diagnosis.* We pass over symptomatology and semeiology to notice this, the most important domain of diagnosis. A case of disease of the heart in a male, aged 26 years, is detailed at length, in illustration of Dr. Siebert's method. We wish we could transfer it to our pages for the benefit of those who cram the weekly journals with their crude, unmeaning, and useless histories. The facts of the case are systematically detailed under different heads: Firstly, the man's medical biography—the *anamnesis* is given—the commencement of the disease, then its course hitherto, with the patient's present condition, under the subdivisions of external appearances, symptoms implicating the circulation, respiration, digestion, and feelings of the patient. The whole phenomena of the disease being ascertained, they are next studied, and their relative value estimated. The anatomical signs of hypertrophy are compared with the functional disturbance in the heart's action, and these with the other symptoms, the objective and subjective, the local and positive, the idiopathic and sympathetic, deutero-pathic and trito-pathic, &c.

The second step is the arrangement of the symptoms into a known form of disease. This may be done by the processes of "subsumption," and comparison and exclusion. In the former a chain of inferences less and less general are made from the phenomena until a special inference, and the special pathology of the disease is deduced. The process of "subsumption" inquires what the disease must be: the process of comparison and exclusion shows what it cannot be. The last step is the consideration of the individuality of the patient, the amount of health remaining to him, and the relation of the disease to the weather and the epidemic "genius." The portion of special diagnosis contained in this, the first volume, comprises the exploration of the heart. The mechanism of the heart's movements, and the nature and causes of its sounds are discussed at length. We must defer, however, the notice of this part to a future opportunity.

---

